# 1Q12 Loci Movement in the Interphase Nucleus Under the Action of ROS Is an Important Component of the Mechanism That Determines Copy Number Variation of Satellite III (1q12) in Health and Schizophrenia

**DOI:** 10.3389/fcell.2020.00386

**Published:** 2020-06-05

**Authors:** Marina Sergeevna Konkova, Elizaveta Sergeevna Ershova, Ekaterina Alekseevna Savinova, Elena Mikhailovna Malinovskaya, Galina Vasilievna Shmarina, Andrey Vladimirovich Martynov, Roman Vladimirovich Veiko, Nataly Vyacheslavovna Zakharova, Pavel Umriukhin, Georgy Petrovich Kostyuk, Vera Leonidovna Izhevskaya, Sergey Ivanovich Kutsev, Natalia Nikolaevna Veiko, Svetlana Victorovna Kostyuk

**Affiliations:** ^1^Federal State Budgetary Scientific Institution, Research Centre for Medical Genetics, Moscow, Russia; ^2^N.A. Alekseev Clinical Psychiatric Hospital No 1, Moscow Healthcare Department, Moscow, Russia; ^3^I.M. Sechenov First Moscow State Medical University of the Ministry of Health of the Russian Federation, Moscow, Russia; ^4^P.K. Anokhin Institute of Normal Physiology, Moscow, Russia

**Keywords:** CNVs, satellite III, rDNA, schizophrenia, 1q12

## Abstract

**Introduction:** Genome repeat cluster sizes can affect the chromatin spatial configuration and function. Low-dose ionizing radiation (IR) induces an adaptive response (AR) in human cells. AR includes the change in chromatin spatial configuration that is necessary to change the expression profile of the genome in response to stress. The 1q12 heterochromatin loci movement from the periphery to the center of the nucleus is a marker of the chromatin configuration change. We hypothesized that a large 1q12 domain could affect chromatin movement, thereby inhibiting the AR.

**Materials and Methods:** 2D fluorescent *in situ* hybridization (FISH) method was used for the satellite III fragment from the 1q12 region (f-SatIII) localization analysis in the interphase nuclei of healthy control (HC) lymphocytes, schizophrenia (SZ) patients, and in cultured mesenchymal stem cells (MSCs). The localization of the nucleolus was analyzed by the nucleolus Ag staining. The non-radioactive quantitative hybridization (NQH) technique was used for the f-SatIII fragment content in DNA analysis. Satellite III fragments transcription was analyzed by reverse transcriptase quantitative PCR (RT-qPCR).

**Results:** Low-dose IR induces the small-area 1q12 domains movement from the periphery to the central regions of the nucleus in HC lymphocytes and MSCs. Simultaneously, nucleolus moves from the nucleus center toward the nuclear envelope. The nucleolus in that period increases. The distance between the 1q12 domain and the nucleolus in irradiated cells is significantly reduced. The large-area 1q12 domains do not move in response to stress. During prolonged cultivation, the irradiated cells with a large f-SatIII amount die, and the population is enriched with the cells with low f-SatIII content. IR induces satellite III transcription in HC lymphocytes. Intact SZ patients’ lymphocytes have the same signs of nuclei activation as irradiated HC cells.

**Conclusion:** When a cell population responds to stress, cells are selected according to the size of the 1q12 domain (the f-SatIII content). The low content of the f-SatIII repeat in SZ patients may be a consequence of the chronic oxidative stress and of a large copies number of the ribosomal repeats.

## Introduction

Repetitive elements comprise two-thirds of the human genome (de Koning et al., 2011). It is known that CNVs could cause inherited diseases in the absence of coding-sequence alterations (Freeman et al., 2006; Redon et al., 2006; Henrichsen et al., 2009; Conrad et al., 2010; Brahmachary et al., 2014; Jackson et al., 2018; Monlong et al., 2018). Tandem repeats in human genome are organized in a head-to-tail orientation and are characterized by increased instability with a pronounced quantitative polymorphism (Warburton et al., 2008; Brahmachary et al., 2014; Black and Giunta, 2018; Hannan, 2018; Lower et al., 2018). The rising roles of satellite tandem repeats in genome organization and disease development were suggested (Iafrate et al., 2004; Sebat et al., 2004; Dumbovic et al., 2017). In our previous studies, we described the CNVs of two tandem repeats in human blood leukocytes: ribosomal repeat (Chestkov et al., 2018a; Malinovskaya et al., 2018) and satellite III fragment (f-SatIII), localized in the largest heterochromatin region 1q12 of the first chromosome (Ershova et al., 2019a, c).

f-SatIII (1.77-kb fragment) from satellite III (Cooke and Hindley, 1979) is an AT-rich repeat (with 64% AT pairs). The human genome contains approximately ∼20 pg f-SatIII/ng DNA. In natural human aging, we observed a significant disproportion in the content of f-SatIII in blood leukocytes of the different individuals. We also observed the f-SatIII content disproportion in DNA samples of people working with the sources of IR (Ershova et al., 2019c). The cells of the same strain and of the same body tissue differ significantly in the f-SatIII content (Ershova et al., 2019c, a).

Ribosomal repeat (rDNA) is localized on acrocentric chromosomes and consists of a transcribed region that includes three rRNA genes (18S, 5.8S, and 28S) and a non-transcribed spacer. In the nucleus, rDNA forms the nucleolus: a special structure where rDNA transcription occurs and the initial stages of ribosome biogenesis are realized. The rDNA-transcribed region contains an unusually low number of AT pairs (28%). The human genome, on average, contains 400 copies of the ribosomal repeat or ∼5 pg of rDNA/ng of total DNA. In contrast to the f-SatIII repeat, in the older age group, there is a significant narrowing of the rDNA CN range and the coefficient of variation decreases (Malinovskaya et al., 2018).

Analysis of rDNA and f-SatIII repeat CNVs in the human blood leukocytes earlier revealed an interesting effect in SZ patients. The SZ patients have significantly more rDNA copies than HC (Veiko et al., 2003; Chestkov et al., 2018a). In contrast, the f-SatIII repeat content (or 1q12 size) in the SZ patients’ leukocytes is lower compared to the HC (Kosower et al., 1995; Ershova et al., 2019a). The mechanism regulating the f-SatIII content in health and SZ remains unknown.

Schizophrenia is a mental illness found in ∼1% of the population with 70–80% heritability (Cardno et al., 1999). SZ patients during an exacerbation of the disease experience severe social and emotional stress (Howes and Murray, 2014). Oxidative stress and declined antioxidant statuses in the brain and peripheral tissues of the SZ patients have been reported. Different mechanisms of oxidative stress in SZ have been proposed (Barron et al., 2017; Maas et al., 2017; Patel et al., 2017). However, regardless of the cause, the result is important: in the SZ patients during an exacerbation of the disease, the level of ROS is increased.

Previously, we noticed that the response of SZ patients’ leukocytes to endogenous oxidative stress in some parameters is very similar to the response of healthy cells to the low-dose IR.

For example, the cells of the unmedicated SZ patient as well as the cells exposed to IR increase the mtDNA amount (Chestkov et al., 2018b). The level of the lymphocyte DNA damage in SZ patients is comparable with the DNA damage of the nuclear workers. In the lymphocytes of ∼30% of SZ patients, we observed DNA damage response, which is a typical response of human cells to IR (Korzeneva et al., 2015; Ershova et al., 2017). We also observed very similar changes in the composition of cfDNA in SZ patients and irradiated nuclear workers. In both cases, cfDNA accumulated the easily oxidized GC-rich fragments (GC-DNA), characterized with a pronounced biological activity (Korzeneva et al., 2016; Ershova et al., 2019b, 2020). *In vitro* experiments have shown that GC-DNA stimulates the expression of NOX family enzymes in human cells, in particular the NOX4, which catalyzes the hydrogen peroxide synthesis on the cell surface and in the mitochondria. GC-DNA stimulates the large amounts of proinflammatory cytokines synthesis in human lymphocytes (Speranskii et al., 2015). Thus, GC-DNAs accumulating in cfDNA of irradiated people and SZ patients may be one of the sterile inflammation causes, which is often observed both during irradiation and in SZ.

We found that ∼40% of the irradiated people have significantly reduced f-SatIII content compared to non-irradiated people of the same age. We also observed that the f-SatIII content decreased in the cultured HSFs under oxidizing agent Cr(VI) (Ershova et al., 2019c). All these facts suggest that there is a common mechanism leading to the f-SatIII repeat content decrease in the healthy cells under oxidative stress induced by environmental factors and in the cells of SZ patients during the disease exacerbation.

Moderate ROS levels are known to stimulate an AR in the human cells. AR increases the cells’ resistance to stress (Sokolov and Neumann, 2015; Sisakht et al., 2020). We have shown earlier that an important component of the AR is the chromatin spatial configuration change. We used the 1q12 loci transposition in interphase nuclei from the periphery to the center as a marker of chromatin configuration change. The change in the f-SatIII (1q12) position in the nucleus under the stresses was found in a number of our studies (Spitkovskii et al., 2003; Veiko et al., 2006; Ermakov et al., 2009a, b, 2011, 2013). The cells that, for various reasons, did not change the 1q12 localization in response to IR frequently died during the cultivation (Spitkovskii et al., 2003; Ermakov et al., 2009b).

It can be expected that the 1q12 locus sizes (f-SatIII content) will be important for the realization of the chromatin spatial configuration necessary for AR. The cells with a very large 1q12 loci, possibly, may not be able to chromatin rearrangement due to steric obstacles. Such cells should die first in chronic stress conditions. In this case, the population should accumulate the cells with small 1q12 loci sizes, and a decrease in the f-SatIII content should be found in an isolated DNA.

To test this hypothesis, we analyzed the response of human cultured lymphocytes and MSCs to low doses of IR. In addition, lymphocytes isolated from the blood of the SZ patients in acute psychosis were analyzed. As a result, we have shown that the response to the stress and proliferative stimuli associated with the 1q12 loci movement in the nucleus is not realized in the cells with a large 1q12 loci size.

## Materials and Methods

### SZ Patients and Healthy Volunteers

The study included 50 drug-naive patients inhabiting Moscow (men aged, 25–47 years). Patients were hospitalized in connection with exacerbation of SZ in N.A. Alexeev Clinical Psychiatric Hospital N^$\underline{\text{o}}$^1. Patients were diagnosed with paranoid SZ according to the Diagnostic and Statistical Manual of Mental Disorders, Fourth Edition (DSM-IV) criteria. The control group of the volunteers consisted of 42 men of the same age.

#### The Patients Consent to the Various Analyses Performed

The investigation was carried out in accordance with the latest version of the Declaration of Helsinki and was approved by the Regional Ethics Committees of RCMG, CPH1, and MHRC. All participants signed an informed written consent to participate after the procedures had been completely explained.

### Isolating of DNA From the Leukocytes

Five milliliters of blood was collected from the peripheral vein with a syringe flushed with heparin (0.1 ml/5 ml blood) under strict aseptic conditions. The leukocytes were isolated from 5 ml of blood by the method of Boyum (1968). To isolate DNA, we used the standard method described in detail earlier (Chestkov et al., 2018a). The DNA quantification is performed fluorimetrically using the PicoGreen dsDNA quantification reagent by Molecular Probes (Invitrogen, CA, United States). The DNA concentration in the sample is calculated according to a DNA standard curve. We use EnSpire equipment (Finland) at excitation and emission wavelengths of 488 and 528 nm, respectively.

### Non-radioactive Quantitative Hybridization

The NQH method for f-SatIII and rDNA repeats determination was specified in details previously [Ershova et al., 2019c (Supplement), (2019c) (Supplement)]. We used this method without modifications. Relative standard error for NQH was only 5 ± 2%. The main contribution to the overall error of the experiment is made by the step of isolating DNA from the leukocytes. The total standard error was 11 ± 7%.

#### The DNA Probe

f-SatIII probe was a 1.77-kb cloned *Eco*RI fragment of human satellite DNA (Cooke and Hindley, 1979) labeled with bio-11-dUTP by nick translation. Dr. H. Cook (MRC, Edinburgh, United Kingdom) kindly supplied the human chromosome lql2-specific repetitive satellite DNA probe pUC1.77.

### Cell Culture

Lymphocytes were isolated by centrifugation in the Ficoll-urography system (Paneco, Russia) from heparinized peripheral blood of men. Lymphocytes were transferred to a culture medium containing Hanks’ solution, 1 mM HEPES (Fluka), and 10% fetal calf serum (HyClone, United States).

Mesenchymal stem cells (MSC-2303) were obtained from adipose tissue (Loseva et al., 2012). MSCs were cultured in F10 (Invitrogen) complemented with 20% fetal bovine serum (FBS), 2 mM glutamine, 10 mM HEPES, 100 U/ml penicillin, 100 mg/ml streptomycin, 10^–6^ M dexamethasone, and 2.5 ng/ml basic fibroblast growth factor (FGF) (Sigma–Aldrich).

### Irradiation of the Cells and Incubation With Hydrogen Peroxide

The cells were irradiated at 20°C on the pulsed roentgen radiation unit ARINA-2 (Spectroflash, Russia). The amplitude of voltage on the X-tube was 160 kV, peak energy in the radiation spectrum was 60 keV, and dose rate amounted to 0.16 Gy/min. After irradiation, the cells were incubated for 3 h at 37°C. H_2_O_2_ (30% solution) was added to the culture medium of lymphocytes at a concentration of 10 μM for 3 h at 37°C.

### Preparation of Cellular Samples

The lymphocytes were washed with phosphate-buffered saline (PBS), subjected to hypotonicity (0.075 M KCl solution) and then were fixed with MAA on glass slides. The MSCs in slide flasks were washed with PBS. The slides were removed and placed for 10 min into a cold fixation solution MAA. Having repeated the procedure three times, the slides were dried and subjected to 2D FISH. A part of the preparation after 10 days was stained with silver nitrate.

The description of the fixing method selection is provided in the Supplementary Material. Three reasons to choose MAA (2D FISH) were the following: (1) the same cellular response (1q12 loci transposition) to IR observed in 2D and 3D FISH experiments; (2) higher FISH 1q12 detection efficiency for MAA-fixed lymphocytes; and (3) inapplicability of the Ag-staining method for cells fixed with 3.7% formaldehyde. Previously, other authors have shown that changes in 2D FISH chromosomes topology correlate with 3D FISH topology (Croft et al., 1999; Skalníková et al., 2000).

### Fluorescent *in situ* Hybridization

Before the hybridization, the slides were treated with RNAse A (100 μg/ml). For the hybridization, the protocol and solutions from Abbott Laboratories (Abbott Laboratories, Abbott Park, IL, United States) were used. Hybridization was carried out in the thermostat ThermoBrite (StatSpin, United States) at 42°. Lymphocyte nuclei were stained with PI.

f-SatIII FISH probe was a 1.77-kb-cloned *Eco*RI fragment of human satellite DNA (Cooke and Hindley, 1979). Labeling of plasmid pUC1.77 was performed by nick translation using CGH Nick Translation Kit (Abbott Molecular) under the manufacturer’s protocol with slight modification. Solutions of plasmid DNA (3 μg/μl) were labeled with SpectrumGreen. In the reaction mix, 50% of the deoxythymidine triphosphate (dTTP) was substituted with the labeled deoxyuridine triphosphate (dUTP). About 20% of the fluorescent-labeled nucleotide was incorporated into the DNA, while unincorporated nucleotides were removed by ethanol precipitation. The fragment size was in 300–3000-bp range as determined by electrophoresis in 1% agarose.

### Activity of the Nucleolus

Fixed cells were stained with silver nitrate (Howell and Black, 1980). In each experiment, 150 cells were scanned on photopanels.

### Image Analysis

**FIGURE 1:**
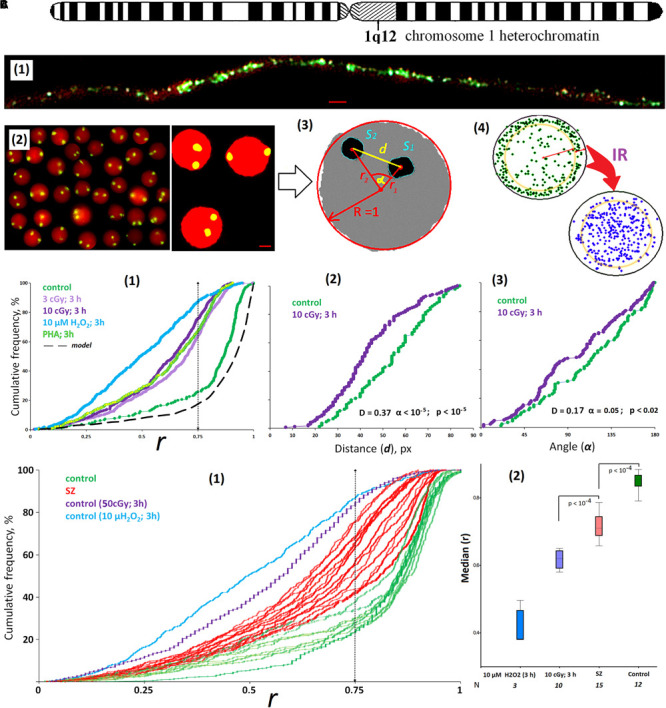
ROS induces the movement of 1q12 loci from the membrane into the nucleus. **(A) (1)** Fiber-FISH chromatin 1q12 analysis with the f-Sat III probe PUC1.77. Repeat clusters of f-SatIII (green) alternate with other repeats (red, PI) of a given region of the chromosome 1. **(2)** The example of the FISH result obtained for the control cells. 1q12 loci are represented in the nucleus (red, PI) by two fluorescent signals (green). Gallery of the cells was formed from multiple photos. Bars, 5 μm. **(3)** Example of the nucleus image analysis. Image processing includes determining the center of gravity of the FISH signal, the radius vectors of the signals (*r*1 and *r*2), the distance and angle between the signals (*d* and α), the area of the FISH signals (*S*1 and *S*2), and radius *R* and area *S* of the nucleus. The radius vector *r* is normalized to the value of the radius of the nucleus *R* and varies from 0 (center of the nucleus) to 1 (surface of the nucleus). **(4)** The total arrangement of FISH signals on the plane in the control sample (green) and the irradiated sample (violet). In the control nuclei, signals with *r* > 0.75 are more common. In irradiated nuclei (10 cGy, 3 h), signals with *r* < 0.75 are more common. **(B) (1)** Cumulative histograms of the frequency distribution of the FISH signals by the radius vector *r* (0: center of the nucleus) for intact, irradiated (3 and 10 cGy), H_2_O_2_-treated, and PHA-stimulated G0 human lymphocytes. Dotted line, 3D–2D simulation under the assumption that FISH signals are located on the surface of a flattened sphere modeling the cell nucleus located on the slide. *Note:* distributions of the *r* values for irradiated HC (3 and 10 cGy) differ significantly from non-irradiated HC: *D* = 0.51, α < 10^–34^ (Kolmogorov–Smirnov); *p* < 10^–50^ (*U*-test). **(2,3)** The distance and angle between the two FISH signals for intact and irradiated (10 cGy) human lymphocytes. **(C) (1)** Cumulative histograms of the frequency distribution of the FISH signals by the radius vector *r* for HC (*N* = 10) and SZ groups (*N* = 15). **(2)** The values of the medians of the radius *r* for the HC group, for irradiated or H_2_O_2_-treated HC cells, and for the SZ cells. Note: Before the FISH nuclei were treated with RNase A.

Cell images were obtained using the AxioScope A1 microscope (Carl Zeiss) with 40 × and 100 × 1.3 lens. To analyze nucleus images after 2D FISH and Ag staining of the NORs, we used two programs: (1) the commercial Carl Zeiss program (Zen 2.6. Blue edition + modules Image Processing and Image Analysis); (2) “A computer program for determining the localization and relative position of chromosome sites in the interphase nuclei of eukaryotic cells (Ellipse)”; the program is registered in the Russian Federation register (No. 2019661442). The Zen 2.6 application translates real signals (spots) and nucleus multiple color images into a schematic image where the nucleus, signals, and background are stained in three different colors [Figure 1A(2)]. The Ellipse program was described earlier (Ermakov et al., 2011). For each schematic image, it defines the following parameters: the nucleus center coordinates; FISH or Ag signal (spots) density distribution on the *X*- and/or *Y*-axis; the parameters associated with the spots density distribution analysis across sectors; the distance from the cell center to the spot center (*Ri*); the angle between the radii R1(FISH) and R2(FISH); the radius of the nucleus *R*; the distance between the centers of spots (*d*); spots area (*Si*); and the nucleus area (*Sn*). An example of the lymphocyte nucleus analysis (a circle in the cross section) is shown in Figures 1A(3,4).

An example of the MSC nucleus analysis is shown in Figure 5A. The shape of the MSCs nuclei may be approximated to a geometric figure—ellipsoid in the cross-section of which lies an ellipse. The program “Ellipse” makes it possible to determine the absolute coordinates of point signals on the plain and values of the greater and smaller axes of the ellipse (a and b). By the affine conversions (rotation of the axes, transposition of the origin of coordinates, and normalization of coordinates of the signal to the axes of the ellipse), the data are transferred to the scheme shown in Figure 5A. Alterations in the position of hybridization signals were tested along two parameters: the normalized radius vector of the labels (*r*) and distance between signals (*d*). The parameter *a*/*b* ≥ 1 reflects an alteration in the shape of the nucleus, while its decrease suggests that it assumes a more spherical shape.

The findings are represented as histograms of the frequency distribution of the hybridization signal of 1q12 (or Ag-NORs) by the normalized radius vector (*r* = *Ri*/*R*) or by the normalized S^FISH^ (S^AgNOR^) of the cell nucleus. For each distribution, we used the data obtained from 100–500 cells.

#### 3D–2D Modeling

The lymphocytes immobilized on the glass are similar in shape to a flattened sphere, so we used a model that includes a mathematical sphere description. It allows placing the points that mimic the labeled chromosome regions in a desired way within the sphere and orthogonally project their position on the plane. In each projection act, the sphere is randomly oriented and flattened along the *Z*-axis (the sphere radius on the *Z*-axis may change). On the projection, the distances of each point from the sphere projection center and the angles (distances) between the points relative to the center were determined. The obtained parameter distributions measured in experiments were compared with the parameters set in the sphere space (Figure 1B, dotted curve). The 3D image was transformed into a 2D image by means of an internal algorithm (RCMG, Moscow, Russia).

### Quantification of RNA *SATIII* Levels

Total RNA was isolated from cells using the RNeasy Mini Kit (Qiagen, Germany). After the treatment with DNAse I, RNA samples were reverse transcribed by the Reverse Transcriptase Kit (Sileks, Russia). The expression profiles were obtained using quantitative reverse transcriptase polymerase chain reaction (qRT-PCR) with SYBRgreen PCR MasterMix (Applied Biosystems). The housekeeping gene *TBP* was evaluated as reference gene. The RNA levels were analyzed in several independent experiments using the StepOne Plus (Applied Biosystems); the technical error (%CV) was ∼2%. All PCR products were run in the polyacrylamide gel (PAGE) to confirm their size. The following primers (Metz et al., 2004; Enukashvily et al., 2007) were used (Sintol, Russia):

HS3-1 (F: 5′AGTCCATTCAATGATTCCATTCCAGT-3′; R: 5′GAATAAAATTGATTGAAATCATCATCC-3′)
HS3-9 (F: 5′AATCAACCCGAGTGCAATC-GAATGGAA TCG3′; R: 5′TCCATTCCATTCCTGTACTCGG 3′).

### Statistical Analysis

All the findings reported here were reproduced at least two times as independent biological replicates. The significance of the observed differences was analyzed using the non-parametric Mann–Whitney *U*-test (*p*) and Kolmogorov–Smirnov statistics (*D* and α). Data were analyzed with StatPlus2007 professional software^1^ and Statistica [TIBCO Software Inc. (2018), version 13^2^ ]. All *p*-values were two-sided and considered statistically significant at *p* < 0.01.

## Results

### Localization of 1q12 Loci in Human Lymphocyte Interphase Nuclei

The f-SatIII repeat analyzed by the 2D FISH method is part of the largest heterochromatin block (1q12) in the human nucleus (Figure 1A). The blocks of f-SatIII tandem repeats are dispersed in the 1q12 region, alternating with other genome repeats, which are clearly visible using spray-FISH method [Figure 1A(1)]. In the lymphocyte nucleus, the f-SatIII repeat is localized in two regions corresponding to the location of two first chromosome homologs. These regions are detected by the FISH method as two fluorescent signals [FISH signals, Figure 1A(2)]. In lymphocytes, the position of the FISH signal in the projection plane (circle) depends on how the nucleus is located on the slide during the sample preparation. Image processing includes determining the gravity center of the signal, the signal radius vector (*r*1 and *r*2) value, the distance and angle between the signals (*d* and α), the signal area, and the radius and the nucleus area [Figure 1A(3)]. The radius vector *r* is normalized to the value of the nucleus radius and changes from 0 (nucleus center) to 1 (nucleus surface). Figure 1A(4) summarizes the data of the HC lymphocyte nuclei hybridization signal (green dots) analysis. Most signals are located in the area corresponding to *r* values > 0.75. Computer modeling translation of 3D images into 2D shows that the signal distribution in the projection shown in Figure 1A(4) corresponds to the location of these signals near the surface of the sphere simulating the lymphocyte nucleus. Thus, in the healthy people lymphocyte nuclei, the 1q12 loci detected by the f-SatIII DNA probe are located near the nuclear envelope.

#### ROS Induce the 1q12 Loci Movement From Periphery to the Center of the Nucleus

Figure 1B(1) shows the f-SatIII localization in the HC lymphocyte nucleus; the data are presented in the form of a cumulative distribution of the normalized radius vector *r* (green curve). In control lymphocytes, the *r* distribution is similar to the distribution obtained by modeling (black dotted curve). In the model, it was assumed that the signals are located exclusively on the surface of the flattened sphere that simulates the lymphocyte nucleus. Low-dose IR or hydrogen peroxide (10 μM, 3 h) significantly changes the 1q12 loci position in the nucleus [Figures 1A(4),B(1)]. In response to stress, 1q12 loci move from the perimembrane region (*r* > 0.75) deep into the nucleus and converge with each other [Figures 1B(2,3)]. A similar 1q12 loci movement is also observed when a proliferative stimulus PHA is applied to the lymphocytes [Figure 1B(1)].

Lymphocytes isolated from the SZ patients’ blood differ from that of the control by 1q12 loci localization inside the nucleus [Figure 1C(1), red]. Figure 1C(2) shows the *r* median values determined for control irradiated and non-irradiated lymphocytes and SZ patients’ lymphocytes. The patients’ lymphocytes occupy an intermediate position between the control non-irradiated and irradiated lymphocytes. For some patients, the 1q12 localization coincided with the locus localization in the lymphocytes irradiated with 3 and 10 cGy doses. It can be assumed that in the patients’ organisms in acute disease stage, the lymphocytes are exposed to oxidative stress, comparable in intensity to the low-dose IR effects.

#### The 1q12 Loci Movement in Response to Stress Depends on the Locus Size

**FIGURE 2:**
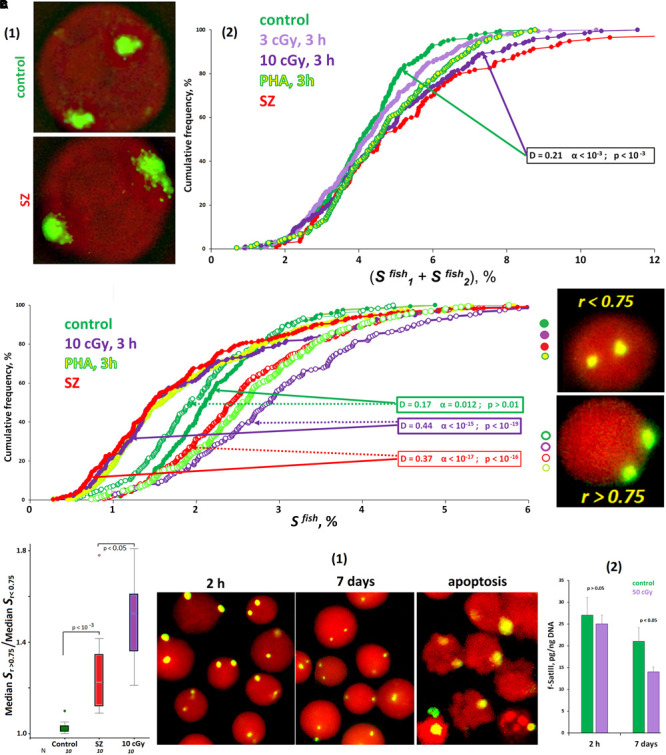
ROS affect the size of f-SatIII repeat in the cells. **(A)** The area of FISH signals in the nuclei of activated lymphocytes (SZ or IR) is increased. **(1)** The photo of the control nucleus and the nucleus of the activated SZ cell. **(2)** Cumulative histograms of the frequency distribution of the FISH signals by the total square S^FISH^ for intact, irradiated (3 and 10 cGy), and PHA-stimulated G0 human lymphocytes and SZ lymphocytes. The signal area increases in about half of the nuclei of the activated cells. **(B)** Cumulative histograms of the frequency distribution of the FISH signals by the square S^FISH^ for intact, irradiated (10 cGy), PHA-stimulated HC lymphocytes, and SZ lymphocytes. Each sample of FISH-signals was divided into two fractions: signals with *r* > 0.75 and *r* < 0.75. The data of comparison of two fractions by the Kolmogorov–Smirnov and Mans–Whitney methods are presented. In the control cells, the two fractions do not differ in the size of the signal areas. In the samples of activated lymphocytes, there is a disproportionation of the cells in terms of f-SatIII repeat areas, depending on r values. **(C)** The values of the ratio median S^FISH^ (*r* > 0.75)/median S^FISH^ (*r* < 0.75) for the HC cells, for irradiated HC cells, and for the SZ cells. **(D)** Prolonged cultivation of irradiated lymphocytes reduces the number of cells with a high f-SatIII repeat content. **(1)** Photos of the cell nuclei after 2 h and 7 days after irradiation with a dose of 50 cGy. **(2)** Change in the repeat content in the DNA of irradiated and unirradiated HC lymphocytes during cultivation determined by method NQH. *Note:* Before the FISH nuclei were treated with RNase A.

The signal area (S^FISH^) in the control cells varies significantly (from 2 to 8% of the nucleus projection area on the plane). That variability may be associated with different f-SatIII content in cells of the same sample, as well as with different chromatin compaction degrees. In the irradiated HC lymphocytes and in the SZ patients’ lymphocytes, the signal areas increase slightly in about a half of the cells [Figure 2A(2)]. The signal form indicates chromatin decondensation in activated cells [Figure 2A(1)]. At the same time, the average f-SatIII repeat content determined by the NQH method does not change in the cell population for 3 h after IR exposure (*p* > 0.05).

Furthermore, we analyzed the dependence of the S^FISH^ signal area on the radius vector *r* value (Figure 2B). In the control cells, we found no differences in the signal area in cells with *r* > 0.75 and *r* < 0.75. However, in activated lymphocytes, there are significant differences in signal areas, characterized by different *r* values. Signals with *r* < 0.75 occupy a much smaller area than signals with *r* > 0.75. Differences in the signal areas of the two groups are maximal for irradiated cells. The SZ patients’ lymphocytes also differ significantly from the control by that factor (Figure 2C). Thus, irradiation and PHA stimulation of healthy donors’ lymphocytes induces the 1q12 loci movement, which occupies a relatively small volume, deep into the nucleus. Loci of large size remain close to the membrane of the nucleus. Lymphocytes of SZ patients subjected to oxidative stress *in vivo* are also characterized by a disproportion of the signal area depending on the signal location.

#### High f-SatIII Content Lymphocytes Are Less Resistant to ROS

We analyzed the f-SatIII content change in the irradiated (50 cGy) lymphocytes DNA during longer cultivation (7 days) after irradiation (Figure 2D). During cultivation, some cells die and have signs of apoptosis and necrosis [Figure 2D(1), apoptosis]. The average f-SatIII repeat content in the isolated DNA of irradiated lymphocytes, determined by the NQH method, is reduced by almost two times compared to the cultivation start [Figure 2D(2)]. At the same time, the population mainly contains the cells with only small 1q12 loci sizes [Figure 2D(1), 7 days]. Thus, in response to oxidative stress, the population cells are selected by the f-SatIII repeat content. Low repeat containing cells have an advantage. Since this repeat is distributed throughout the 1q12 site, it may be assumed that, predominantly, cells with large 1q12 loci occupying a large nucleus volume die in response to the stress.

#### Satellite III Transcription in Lymphocytes in Response to ROS

**FIGURE 3:**
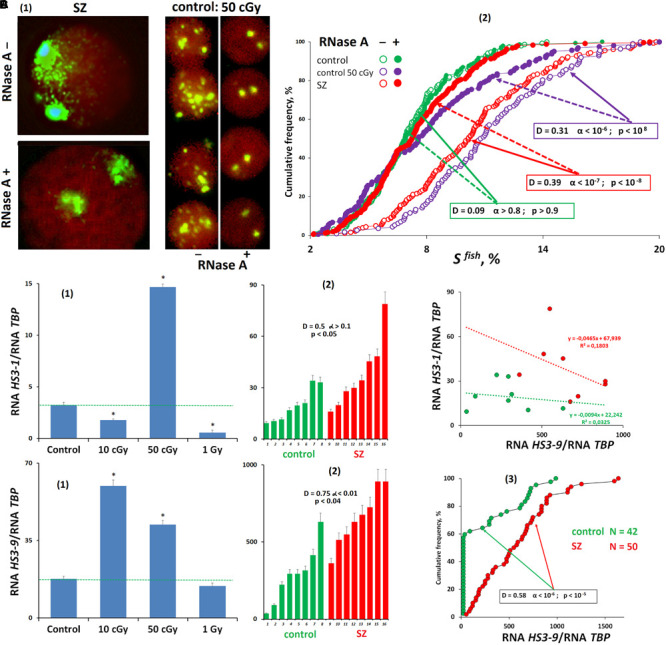
Transcription of satellite III in human cells. **(A)** Processing the nuclei with RNase A reduces the FISH-signal area. **(1)** Examples of the nuclei of SZ patients and nuclei of irradiated HC cells treated and untreated with RNase A before the FISH. **(2)** Cumulative histograms of the frequency distribution of the FISH signals by the square S^FISH^ for treated and untreated with RNase A intact HC, irradiated HC (50 cGy, 48 h), and SZ lymphocytes. **(B)** Transcription of f-SatIII DNA (HS3-1). **(1)** Irradiated lymphocytes (72 h after exposure). **(2)** White blood cells of SZ and HC groups. **(C)** Transcription of satellite III located on chromosome 9 (HS3-9). **(1)** Irradiated lymphocytes (72 h after exposure). **(2,3)** White blood cells of SZ and HC groups. **(D)** Dependence of the amount of RNA *HS3-1* on the amount of RNA HS3-9 for two groups.

Comparing the FISH-signal areas during hybridization of lymphocyte nuclei treated and untreated with RNase A, we found that the f-SatIII DNA-probe hybridizes not only with DNA but also with RNA (HS3-1). Figure 3A(2) shows a comparison of signal areas for the same lymphocyte population. In control cells, we found no differences in the signal area in the RNase A treated and untreated nuclei. However, in RNase-treated stimulated cells (irradiated control lymphocytes and SZ patients’ lymphocytes), we found a significant total S^FISH^ reduction. This indicates the RNA *HS3-1* contribution in the nuclear DNA with the f-SatIII probe hybridization. The maximum S^FISH^ increase in RNase A-untreated nuclei was found in irradiated lymphocytes (50 cGy) after 72 h of cultivation [Figure 3A(1)].

Reverse transcriptase quantitative PCR (RT-qPCR) was applied to test an assumption about studied fragment transcription under irradiation stress [Figure 3B(1)]. The amount of RNA *HS3-1* significantly changed after 72 h of irradiated lymphocytes cultivation. A small dose (10 cGy) and a large dose (1 Gy) reduced the *HS3-1* RNA amount; the effect was maximal for a 1-Gy dose. A 50-cGy dose increased *HS3-1* RNA by several times.

In the same cells, we also studied a satellite III fragment transcription that is localized on chromosome 9 and is often used to analyze the satellite DNA transcription under stress caused by various factors (Valgardsdottir et al., 2008). In contrast to the f-SatIII fragment from the 1q12 region, the satellite fragment of chromosome 9 is maximally transcribed even under the low IR dose [Figure 3C(1)]. An increase in the dose (50 cGy and 1 Gy) decreases the level of *HS3-9* RNA. The RNA *HS3-9* amount in the cells is several times higher than the *HS3-1* transcript. Thus, the satellite III transcription profile in lymphocytes depends on the location of the satellite on chromosomes and on the stress intensity. Low-dose IR exposures activate transcription of satellite III on chromosome 9.

#### Satellite III Transcription in SZ Patients’ White Blood Cells

We compared the *HS3-1* and *HS3-9* RNA levels in the white blood cells of SZ patients and HCs [Figures 3B(2),C(2,3),D]. The *HS3-9* RNA amount in human white blood cells was an order of magnitude higher than the amount of *HS3-1* RNA. The patients’ white blood cells contained more RNA *HS3-1* and RNA *HS3-9* than the control white blood cells. We found a negative relationship between RNA *HS3-1* and RNA *HS3-9* levels (Figure 3D). It confirms the assumption that *HS3-9* transcription is predominant under weak stresses. Figure 3C(3) shows data on the *HS3-9* RNA amount in the white blood cells of 50 SZ patients and 42 healthy people. In the control group, satellite III transcription was observed only in 40% of the samples. In the group of patients, the satellite transcription was much higher.

Thus, stress in the SZ patient’s organism in acute disease stage is accompanied by an increase in satellite III sequences transcription in blood leukocytes.

### Ribosomal DNA Localization in Human Lymphocytes

**FIGURE 4:**
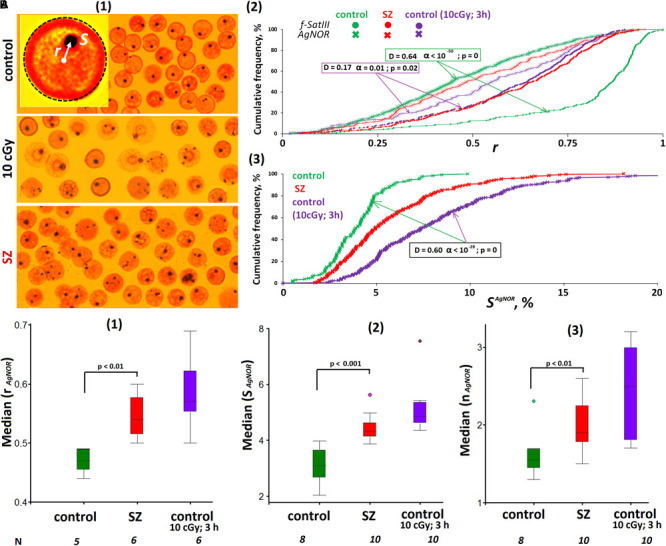
ROS change the location and size of the nucleolus in lymphocyte. **(A) (1)** The example of Ag staining of the nucleolus (AgNOR). Gallery of the nuclei was formed from multiple photos. **(2)** Cumulative histograms of the frequency distribution of the Ag signals by the radius vector *r* (0: center of the nucleus) for intact, irradiated (10 cGy), and SZ lymphocytes. For comparison, the graph shows the data for the FISH signals of the same samples. **(3)** Cumulative histograms of the frequency distribution of the Ag signals by the total square S^AgNOR^ for intact HC, irradiated HC (10 cGy), and SZ lymphocytes. The result is confirmed on five HC and five SZ samples. **(B) (1)** The values of the medians of the radius *r* (AgNOR) for the HC cells, for irradiated HC cells and for the SZ cells. **(2)** The values of the medians of the area S (AgNOR) for the groups. **(3)** The median of the AgNOR copy number for the groups.

Ribosomal repeats in the eukaryotic cell form a special structure—the nucleolus. Various methods may be applied to analyze the rDNA in the lymphocyte nucleus localization: FISH, nucleolus proteins analysis with antibodies, etc. We chose the simplest method using silver nitrate staining of argentophilic nucleolus proteins. This method requires the same nucleus preparation as the 2D FISH method used for f-SatIII fragment analysis. To analyze the image, we used the same algorithm as for the f-SatIII repeat (Figure 4A). The signal radius vector values (dark brown silver spot) and the spot area (S^AgNOR^) were determined.

Figure 4A(2) compares the radius vector value distributions of FISH signals and AgNOR signals in the nuclei of control lymphocytes, irradiated control lymphocytes, and SZ patient lymphocytes. In contrast to f-SatIII, rDNA in the nucleolus of HC are localized in the central nucleus regions—within a sphere with a normalized radius of 0.4–0.5 inside the nucleus (simulation data). In irradiated cells (10 cGy, 3 h), the nucleolus moves from the center of the nucleus (a sphere with a radius of 0.5–0.6), approximately to the same regions of the nucleus where 1q12 loci are localized, which shifted from the nuclear envelope to the center of the nucleus in response to IR. A similar movement of the 1q12 loci and nucleolus was observed in the SZ patients’ lymphocyte nuclei [Figure 4A(2), red curves]. Generalized data for several cell samples are shown in Figure 4B(1). The median values of the Ag-signal radius in activated lymphocytes are significantly higher than in control lymphocytes. The rDNA movement in the nucleolus in activated lymphocytes (irradiated control cells and cells of SZ patients) is accompanied by a significant increase in the total nucleoli area [Figures 4A(3),B(2,3)].

Thus, in activated lymphocytes, there is an increase in the nucleolus area and its displacement to approximately the same nucleus area where the 1q12 loci are localized.

### Localization of f-SatIII and rDNA in the Human MSC

**FIGURE 5:**
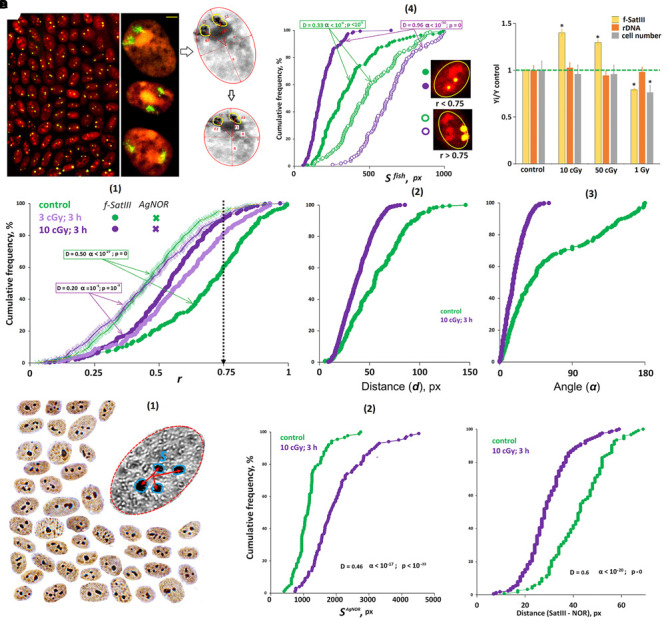
The effect of IR on the localization of 1q12 and nucleolus in MSCs. **(A)** Foto: The example of the FISH result obtained for the MSCs. 1q12 loci are represented in the nucleus (red, PI) by two fluorescent signals (green). Gallery of the cells was formed from multiple photos. The nucleoli contrast in the nucleus. Bars, 5 μm. Colorless drawing: Schematic representation of the MSC nucleus after carrying out affine transformations, where *r*1 and *r*2 are normalized radius vectors of the FISH signals. **(1)** Cumulative histograms of the frequency distribution of the FISH and Ag signals by the radius vector *r* (0: center of the nucleus) for intact and irradiated (3 and 10 cGy) cells. **(2,3)** The distance and angle between the two FISH signals for intact and irradiated (10 cGy) cells. **(4)** Cumulative histograms of the frequency distribution of the FISH signals by the square S^FISH^ for intact and irradiated (10 cGy) MSCs. Each sample of FISH signals was divided into two fractions: signals with *r* > 0.75 and *r* < 0.75. **(B) (1)** The example of the Ag staining obtained for the MSCs. Gallery of the cells was formed from multiple photos. **(2)** Cumulative histograms of the frequency distribution of the Ag signals by the total square S^AgNOR^ for intact and irradiated (10 cGy) MSCs. **(C)** The total distance between the center of the FISH signal and the surface of the nucleolus in the intact and irradiated (10 cGy) MSCs. **(D)** The effect of MSC irradiation on the content of f-SatIII, rDNA, and cell number. Doses are shown in the figure. Irradiated cells were cultured for 72 h.

To confirm the universality of the human cell response to oxidative stress, we analyzed the effect of IR on the cultured adipose tissue MSCs. Subconfluent MSC culture was used for the analysis. The algorithm for analyzing cells with nuclei using the model with a rotation ellipsoid (projection on a plane is an ellipse) was described earlier on the example of endothelial cells (Ermakov et al., 2011). Figure 5A provides examples of cells after the FISH procedure. Nucleoli were determined by Ag staining [Figure 5B(1)]. To analyze the 1q12 loci position and AgNORs, the values of the radius vector *r* normalized to the axes of the ellipse were determined.

Figure 5A(1) shows the histograms reflecting the loci 1q12 localization and AgNORs in subconfluent non-irradiated and irradiated (10 cGy) cells. We found the same response as in the lymphocytes. In irradiated cells, 1q12 loci move from the periphery to the center of the nucleus in the region where the nucleoli are predominantly localized. This reduces the distance and the angle between 1q12 homologs [Figures 5A(2,3)].

Analysis of the FISH-signal areas revealed the same pattern as in the case of lymphocytes: in the central nuclei regions (*r* < 0.75), the 1q12 loci localized with a smaller area [Figure 5A(4)]. Loci with a large area do not change their location in the nucleus in response to stress. The differences observed for control cells appear to be due to the fact that a small portion of the cells are in the G1 phase of the cycle and respond to proliferative stimuli by moving 1q12 from the membrane to the center of the nucleus. In response to the IR, there is also an increase in the total AgNOR area, which indicates an increase in the rDNA volume in the nucleus.

Mesenchymal stem cells turned out to be a convenient object where it is possible to simultaneously analyze the nucleus and 1q12 locus. Using the PI dye (forms complexes with GC-rich DNA sequences) after FISH allows contrasting the nucleolus in the nucleus (Figure 5A, photo). We determined the distance between the FISH signal (green) center and the surface of the nucleoli (red) in non-irradiated and irradiated cells (Figure 5C). The distance between the signals is significantly reduced in the irradiated cells, which indicates that the 1q12 and rDNA loci are converging.

Thus, in irradiated MSCs, 1q12 loci with a small area move from the membrane to the center of the nuclei. In this case, the nucleoli increase in size and approach the 1q12 loci.

### Change in the MSC f-SatIII Content Under IR

The f-SatIII repeat content in cell DNA determined by the NQH method depends on the proportion of cells with high and low repeat content in a population. When low doses of IR are applied (10 cGy, 72 h of cultivation), the repeat content increases in the population, which reflects an increase in the number of cells with an increased f-SatIII content (Figure 5D). Under the large radiation doses (1 Gy), the repeat content reduces, while some cells die. It is logical to assume that the cells with a large f-SatIII repeat number die. In that case, the ribosomal repeat content in the cells does not change. A similar response we observed in a cultured skin fibroblasts population exposed to the different genotoxic agent Cr(VI) concentrations (Ershova et al., 2019c).

## Discussion

### Oxidative Stress Induces the Movement of Two Large Tandem Genome Repeats in the Cultured Human Cells Nuclei

In this study, we investigated how the mutual localization of two large genome tandem repeats in human cell nuclei changes under IR. The satellite domain at chromosome 1q12, detected with the probe for f-SatIII, contains the largest heterochromatin site in the genome, comprising a megabase stretch of satellite II and III DNA repeats. The amount of f-SatIII in DNA appears to reflect the size of this large genome region. The higher is the content of f-SatIII in DNA, the greater is the volume that this part of the genome occupies in the nucleus. We found that the f-SatIII content in the human leukocyte genome varies from 6 to 44 pg/ng of DNA (Ershova et al., 2019c, a), i.e., the size of the 1q12 site may vary several times. It should be mentioned that the cells of one person or one cultured strain also differ in the f-SatIII content. We have shown that HSFs contain cell subpopulations that differ in f-SatIII repeat content by more than three times (Ershova et al., 2019c). The heterogenic cellular f-SatIII content (by 1q12 loci size) is even more pronounced in polyploid cancer cells (Ermakov et al., 2009b; Schwarz-Finsterle et al., 2013 and Supplementary Material) and in cells of various brain regions of the SZ patient (Ershova et al., 2019a). Obviously, such a significant change in the content of a large genome fragment affects the higher order genomic architecture.

In response to stress, these large chromatin fragments move from the surface into the nucleus (Figures 1, 5). The satellite domain (1q12) translocation is the cells’ universal response to various types of stress. We observed this process in human lymphocytes (Ermakov et al., 2009a, 2013; Figure 1), endothelial cells (HUVECs) (Ermakov et al., 2011), MSCs (Figure 5), and cancer cells (Ermakov et al., 2009b; Supplementary Material). Our data on the transposition and convergence of heterochromatin 1q12 loci of homologous chromosomes in response to IR confirm the data of other authors. It has been shown that IR induces instant human-cell homologous chromosomes heterochromatin pairing (Dolling et al., 1997; Abdel-Halim et al., 2004).

The main condition for 1q12 loci movement in response to environmental factors is the presence of ROS in the intercellular environment or on the cell surface (Ermakov et al., 2009a, 2013). The nature of the ROS source is not significant. It may be low-dose IR or hydrogen peroxide [Figure 1B(1)], NOX family enzymes inductors—fragments of cell-free DNA (Ermakov et al., 2013; Ershova et al., 2020) and endogenous stress caused by SZ disease (Figures 1B,C). ROS inhibition with antioxidants blocks the 1q12 movement in the interphase nucleus (Ermakov et al., 2009a).

However, the forces and molecular mechanisms that shape the radial configuration of the 1q12 loci under the ROS action remain largely elusive. Many authors believe that anchoring of chromosomes to the nuclear lamina via LADs at the nuclear periphery is a key regulator of the radial configuration of chromatin. Genome fragments similar to the analyzed 1q12 loci belong to LADs. LADs are gene poor, heterochromatic, and transcriptionally silent. They are typically AT-rich sequences, possess heterochromatin marks like H3K9me3 and H3K9me2, and overlap with the late replicating regions of DNA during S phase (Guelen et al., 2008; Collas et al., 2019; Sivakumar et al., 2019). Dynamic interactions of chromatin with the nuclear lamina-associated protein complexes provide ways of radially repositioning chromatin in the nucleus (Reddy et al., 2008; Solovei et al., 2013; Gordon et al., 2015; Kind et al., 2015). Knockout of the proteins of the nuclear lamina led to condensation of heterochromatin in the nuclear interior (Solovei et al., 2013). Knockdown of the lamina protein emerin resulted in chromosome repositioning inside the nucleus and reduction in H3K9me3 levels and distribution (Le et al., 2016; Ranade et al., 2019). Earlier, we demonstrated an increase in the activity of caspase-3 after irradiation of lymphocytes at a dose of 10 cGy. Caspase-3 activity inhibition abolishes the observed translocations of the 1q12 loci in the irradiated human cells (Ermakov et al., 2009a). One cannot exclude that the protease activity of caspase-3 is necessary for the observed structural rearrangement of chromatin on exposure to IR. Caspase-3 may participate in freeing 1q12 loci from the connection with the nuclear lamina.

Histone modifications might also play a role in shaping chromatin configuration. The treatment of the cells with a histone deacetylase inhibitor resulted in the relocation of the chromatin loci from the nuclear periphery toward the center (Strasák et al., 2009). In addition, it was recently proposed that transcriptional activity of the genome represents the main force that changes the radial chromatin configuration in the nucleus (Cook and Marenduzzo, 2018). Some authors believe that the mechanisms of chromatin configuration change involve a phase separation process, which has been shown to be implicated in the formation of heterochromatin and in driving the transition of euchromatin to heterochromatin (Larson et al., 2017; Strom et al., 2017).

Domain 1q12 transposition is an important component of the adaptive cellular response to oxidative stress induced by IR. The absence of 1q12 displacement is associated with AR block and increased cell death under stronger exposure. Previously, we observed 1q12 displacement block in lymphocytes of breast cancer patients with a *BRCA1* gene mutation (Spitkovskii et al., 2003) and in 1q12 polyploid primary stem cancer cells of the breast tumor (Ermakov et al., 2009b). The primary cancer cell population at the beginning of cultivation contained 70% of cells with a polyploid 1q12 loci set (data are given in the Supplementary Material). In polyploid set cells, 1q12 did not move in response to irradiation. The loci were “bound” to the nucleus membrane. During long-term cultivation, these cells died first, and the population was enriched with cells with a normal 1q12 diploid set, which is in response to irradiation-transposed 1q12 loci from the periphery to the nucleus center.

In a study of a high radiation dose (10 Gy) effect on the triploid by 1q fragment content cells of the Hela cancer line, the authors found an increase in the content of 1q fragment in the nuclei on the fifth day (Schwarz-Finsterle et al., 2013). With longer cultivation, the survival advantage was found in cells with reduced 1q fragments content. One of the reasons for increased survival may be the ability of these cells to proliferate and respond adaptively, in contrast to cells with a high 1q content.

Previously, we considered domain 1q12 translocation in response to low-dose IR only as a marker that reflects a change in the nucleus architecture for genome expression profile modulation in response to the damage. In this paper, for the first time, we analyzed the possible active role of the 1q12 domain size in the process of nucleus architecture changes in stress response. We studied two types of the cells: spherical lymphocyte nuclei, which may be placed randomly on the slide, and ellipsoid MSC nuclei, which occupy a fixed position on the carrier. In both cases, we found a similar effect: in response to ROS, only 1q12 domains of relatively small size moved to the nucleus center from the nuclear envelope (Figures 2, 5).

Ribosomal repeat is also represented in the human genome by a large number of copies. In our sample, the rDNA content ranged from 3 to 11 pg/ng DNA (Chestkov et al., 2018a; Malinovskaya et al., 2018). In the absence of stress in the interphase nucleus, rDNA copies are located compactly in the nucleolus in the central nucleus regions. In response to stress, the area occupied by rDNA may increase several times. We observed an increase in the NOR area in response to stress for human lymphocytes (Ermakov et al., 2009a, 2013) and endothelial cells (Ermakov et al., 2011).

In irradiated cells, two large domains (nucleolus and 1q12 heterochromatin) seemingly move toward each other and are localized in the spherical ring area with a radius of about 0.5–0.6 of the nucleus radius. Some other researchers’ data show the rDNA and 1q12 loci interaction in the interphase nuclei. It was shown that 1q12 regions contribute to the perinucleolar chromatin. During the cell cycle, the heterochromatic band 1q12 is dynamically rearranged with regard to the nucleoli. A relationship between the association of the chromosome 1 pericentromeric region with nucleoli and the nuclear transcriptional activity was suggested (Léger et al., 1994). These facts are confirmed by the other authors, who have shown the emergence of numerous new contacts of rDNA with 1q12 region, under cellular stress (Tchurikov et al., 2019). The nucleolus and 1q12 domain convergence also occurs due to a significant increase in the nucleolus area and the nucleolus’s number (Figure 4). Presumably, the large-volume 1q12 domain will not be able to move from the membrane to the desired nucleus sector, and the chromatin transformations necessary to change the genome expression profile in response to stress are not implemented. If the nucleolus is very large (e.g., the genome contains many rDNA copies) and occupies a large nucleus volume, then the requirement for the 1q12 domain size increases. Only small-sized 1q12 loci will be able to localize in the “right” nucleus sector. Thus, it can be assumed that, in the cell, there is a balance between the sizes of rDNA clusters and the sizes of 1q12 heterochromatin region. Disruption of this balance may lead to abnormal cell functioning.

**FIGURE 6:**
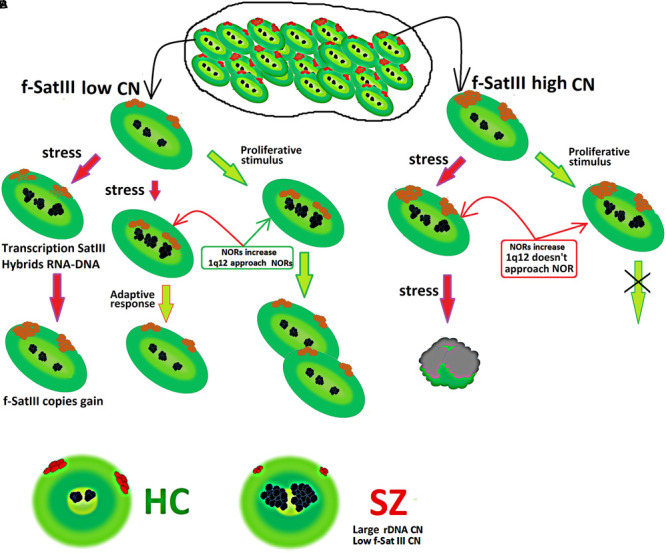
**(A)** Scheme showing a change in the content of f-SatIII in a cell population under stress. A detailed description is given in the text. **(B)** Illustration of f-SatIII and rDNA tandem repeats sizes in HC and SZ groups.

Figure 6A summarizes the facts obtained in the study. Consider a population of cells that are heterogeneous in terms of f-SatIII DNA content. Cells with a low repeat number (small 1q12 domain size) are able to proliferate and develop an adaptive stress response. Both processes require 1q12 movement in the nucleus and bringing it closer to the nucleolus that increases with stress response or a proliferative stimulus. The AR increases the cells’ resistance to stress (Sokolov and Neumann, 2015; Sisakht et al., 2020). Thus, cells with a low f-SatIII content have a large proliferative potential and genotoxic stress resistance (Ershova et al., 2019c).

The transition of satellite III heterochromatin to euchromatin and the activation of the satellite transcription occurs in some cells (Figure 3). This process leads to the f-SatIII content increase in the cell’s DNA (Bersani et al., 2015), and these cells replenish the cells fraction with a large f-SatIII repeat size. Cells with a high f-SatIII content accumulate in the population during natural and replicative aging (Ershova et al., 2019c) and under low IR doses (Figure 5D). Such cells are not able to proliferate and die under more intense exposure being not capable to an AR.

### White Blood Cells of SZ Patients Show a Response Comparable to the Low-Dose IR Effect on Healthy Cells

Schizophrenia is considered as a systemic disorder (Kirkpatrick, 2009). The available biomaterial studies, e.g., of blood leukocytes, helps to understand the possible disease mechanisms (Chan et al., 2011; Lai et al., 2016; Sabherwal et al., 2016; Perkovic et al., 2017). Oxidative stress and declined antioxidant statuses in the brain and peripheral tissues of the SZ patients have been reported (Barron et al., 2017; Maas et al., 2017; Patel et al., 2017).

Analysis of rDNA and f-SatIII CNVs in the blood leukocytes earlier revealed an interesting effect in SZ patients. The patients have significantly more rDNA copies than HC (Veiko et al., 2003; Chestkov et al., 2018a). In contrast, the f-SatIII repeat content in the SZ patients’ leukocytes is significantly lower compared to the HC (Kosower et al., 1995; Ershova et al., 2019a). The results of the present study may explain these facts (Figure 6B).

The lymphocytes isolated from the blood of SZ patients have activation signs typical also for control lymphocytes irradiated with low-dose IR (Figures 1–4). Small-sized 1q12 domains translocated to the central nucleus area; the nucleoli occupied a large area and approached the 1q12 region. SZ patients’ lymphocytes activation has been repeatedly described in a number of papers (Hirata-Hibi et al., 1982; Kloukina-Pantazidou et al., 2010; Uranova et al., 2017). Some authors have described the nucleolus increase (Uranova et al., 2017). It may be assumed that in the patients’ organism oxidative stress chronically stimulates an AR for reparative, antioxidative, and antiapoptotic systems activation. The source of oxidative stress in SZ is not yet reliably determined, but its intensity is comparable to the effect of low-dose IR. Earlier, in a third of SZ patients, we described an AR that allows survival of cells with damaged DNA. We also found a significant increase in cell death in patients, indicated by abnormally high amounts of cfDNA and increased endonuclease blood plasma activity (Ershova et al., 2017, 2019b).

Perhaps, in the patients’ organism, the process of blood cells selection by the f-SatIII repeat content is significantly accelerated in comparison with the control. A similar process was observed during long-term irradiated healthy donor lymphocytes cultivation (Figure 2D). An additional factor of cells selection with only a low f-SatIII amount is the large size of the nucleolus that contains more rDNA than the control cells nucleolus. It is assumed that rDNA in the nucleus stabilizes heterochromatin regions (Paredes and Maggert, 2009). A large rDNA amount shifts the heterochromatin–euchromatin balance toward heterochromatin. It has also been shown that a change in the rDNA clusters size leads to a significant change in the expression profile of many genes located at a significant distance from the rDNA (Paredes et al., 2011).

Thus, the low f-SatIII content in white blood cells DNA of SZ patients may be explained by three reasons:

(1) Large rDNA cluster sizes stabilize 1q12 heterochromatin, reducing the satellite transcription intensity that contributes to f-SatIII content increase.
(2) Chronic oxidative stress induces an AR only in cells with a low f-SatIII content.
(3) Cells with a high f-SatIII content, in which the AR is blocked, are less resistant to damage effects and die.

Processes leading to f-SatIII content (1q12 region size) changes in blood cells also occur in the SZ patients’ brain cells. We have shown that the f-SatIII repeat content varies significantly in eight different brain structures of the SZ patient (Ershova et al., 2019a). Regions with a high f-SatIII repeat content at the same time contained lower amounts of telomeric repeat. The accumulation of brain cells with a high f-SatIII content, apparently, may change the normal functional activity of various brain structures cells.

Further research is needed to explain the combination of high rDNA and low f-SatIII in the genomes of SZ patients. In particular, it would be important to get answers to the following questions:

1. Are very large rDNA CN in the human genome capable of blocking the heterochromatin–euchromatin transition in the 1q12 region that we detect with the f-SatIII probe? It is interesting to compare the RNA SATIII synthesis during response of cells with different combinations of f-SatIII and rDNA CN to replicative aging and genotoxic stress. It is also important to compare RNA SATIII transcription levels in the genomes of SZ patients and control persons with different combinations of f-SatIII and rDNA CN indices.
2. How is the variation in the two repeats content in different human brain cells associated with pathology? What is the difference of repeats content in the brain cells between mentally healthy and SZ suffering people?
3. What other diseases may be associated with a particular f-SatIII and rDNA CN combination? Most likely, it may be some multifactorial diseases. Perhaps, some diseases may manifest (or not manifest) itself only in case of a specific combination of the two repeats content.
4. Does the rDNA content in the human cell genome correlate with the content of other genome satellite repeats that are able to be transcribed?

## Conclusion

When a cell population responds to stress, cells are selected according to the size of the 1q12 domain (according to the content of the f-SatIII repeat). The low content of the f-SatIII repeat in SZ patients may be a consequence of the chronic oxidative stress and of a large copies number of the ribosomal repeats.

## Data Availability Statement

All datasets generated for this study are included in the article/Supplementary Material.

## Ethics Statement

The investigation was carried out in accordance with the latest version of the Declaration of Helsinki and was approved by the Regional Ethics Committees of RCMG, CPH1, and MHRC. All participants signed an informed written consent to participate after the procedures had been completely explained.

## Author Contributions

SVK, SIK, and NV designed the study. NZ and GK examined and selected patients for the study, performed analysis using a scale PANSS, and provided the human blood samples. EE, MK, ES, EM, GS, AM, and VI performed the experiments. RV performed the statistical analysis and created a computer database for SZ and HC groups and programs “Imager 7.0.” and “Ellipse.” SVK, PU, and NV wrote the initial draft and translated the manuscript to English. All the authors participated in critical revision and approved the manuscript before submission.

## Conflict of Interest

The authors declare that the research was conducted in the absence of any commercial or financial relationships that could be construed as a potential conflict of interest.

## Footnotes

1 http://www.analystsoft.com/

2 http://tibco.com

## Abbreviations

AR: adaptive response
cfDNA: circulating cell-free DNA
CNVs: copy number variants
FISH: fluorescent *in situ* hybridization
f-SatIII: the satellite III fragment from the 1q12 region
HC: healthy control
HSFs: human skin fibroblasts
IR: low-dose ionizing radiation
LADs: lamina-associated domains
MAA: methanol:glacial acetic acid (3:1)
MSCs: mesenchymal stem cells
mtDNA: mitochondrial DNA
NQH: non-radioactive quantitative hybridization
PHA: phytohemagglutinin
PI: propidium iodide
rDNA: ribosomal repeat
ROS: reactive oxygen species
SZ: schizophrenia.
