# Supplementary material for: 1Q12 Loci Movement in the Interphase Nucleus Under the Action of ROS Is an Important Component of the Mechanism That Determines Copy Number Variation of Satellite III (1q12) in Health and Schizophrenia
Source: Front Cell Dev Biol. 2020 Jun 5;8:386. doi: 10.3389/fcell.2020.00386 (PMC7346584; doi:10.3389/fcell.2020.00386)
Supplement: Supplementary file 1 [file Table_1.DOCX]

**1S. The choice of the lymphocytes and MSCs nuclei fixation method.**

FISH studies relied on the two main methods of nucleus fixation: fixation with 1.8 – 3.7 % formaldehyde in PBS buffer (BF) with subsequent permeabilization and hypotonic treatment (0.075 M KCL) with subsequent methanol: acetic acid (3:1) fixation (MAA). This methods are known as 3D and 2D FISH (Hepperger et al., 2007; Croft et al. 1999). BF to a lesser extent changes the spatial organization of chromatin in the cell nuclei than MAA. To choose an acceptable analysis method, we compared both cell fixation protocols, described in details previously (Hepperger et al., 2007; Weierich et al., 2003; Croft et al. 1999).

**MSC.** BF was applied to MCS cells as 3.7 % in PBS for 15 min and washed 3×10 min in PBS. Nuclei for FISH were permeabilized for 15 min in 0.5% Triton-X-100 in PBS. After three washes in PBS (10 min each) hybridization mixture was added to wet slides.

In MAA fixation protocols without hypotonic treatment, the MCS cells in slide-flasks were washed with PBS. The slides were removed and placed for 10 min into a cold ( −20°C) fixation solution: methanol/glacial acetic acid (3:1). Having repeated the procedure three times, the slides were dried and subjected to FISH after 5 days.

**Lymphocytes.** The lymphocytes were subjected to hypotonicity (0.3 PBS) and were placed on coverslips coated with polylysine for 40 min at 37°C. Formaldehyde was applied to cells (3.7% in PBS) for 15 min and washed 3×3 min in PBS. Nuclei for 3D FISH were permeabilized for 15 min in 0.5% Triton-X-100 in PBS, immersed in 20% glycerol in PBS for 1 h, and subjected to 5 freeze/thaw cycles with liquid nitrogen. After three washes in PBS (3 min), the cells were incubated 10 min in 0.1 N HCL, washed 3×3 min in 2×SSC, and stored (1 weeks) in 50% formamide/50% 2×SSC. Hybridization mixture was added to wet slides.

In MAA fixation protocols the lymphocytes were subjected to hypotonicity (0.075 M KCl solution) and were then ﬁxed with [methanol:glacial acetic acid (3:1)] using standard procedures.

A part of preparations was stained with silver nitrate 5-10 days later.

Analysis of the 1q12 domains movement under the radiation in lymphocytes and MCS cells showed that the cell fixation method has almost no effect on the final result (the analyzed 1q12 domain position in the nucleus). The fluorescent signals intensity was higher in case of MAA fixation. In contrast to BF fixation, during MAA fixation virtually no nuclei were observed with a single signal or without a signal. Previously, it was shown that changes in the chromosomes topology during 2D FISH correlate with the changes recorded by 3D FISH (Croft et al. 1999; Kosubek et al., 1999).

The BF fixation method proved to be of little use for AgNOR analysis due to a very high non-specific response. In addition to AgNOR, other structures were stained in the cells (figure 1S: Ag-staining of MSCs nuclei). Thus, during the main experiments, the choice of the fixation method was made in favor of MAA. This method allowed analyzing the 1q12 and AgNOR domains in similar nuclei preparations.


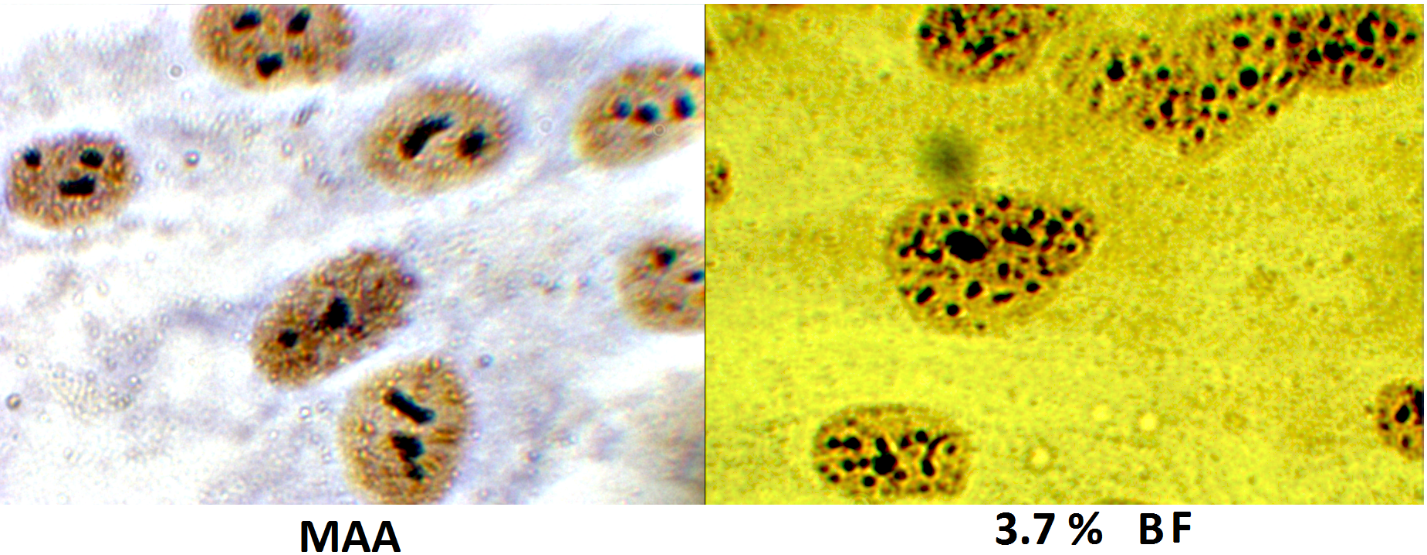


Figure 1S.

**2S. The cancer stem cells response to the IR.**

Experimental data previously published in our study in Russian are presented: [Ermakov, A.V](https://www.ncbi.nlm.nih.gov/pubmed/?term=Ermakov%20AV%5BAuthor%5D&cauthor=true&cauthor_uid=19947515)., [Kon'kova, M.S](https://www.ncbi.nlm.nih.gov/pubmed/?term=Kon%27kova%20MS%5BAuthor%5D&cauthor=true&cauthor_uid=19947515)., [Kostiuk, S.V](https://www.ncbi.nlm.nih.gov/pubmed/?term=Kostiuk%20SV%5BAuthor%5D&cauthor=true&cauthor_uid=19947515)., [Ershova, E.S](https://www.ncbi.nlm.nih.gov/pubmed/?term=Ershova%20ES%5BAuthor%5D&cauthor=true&cauthor_uid=19947515)., [Smirnova, T.D](https://www.ncbi.nlm.nih.gov/pubmed/?term=Smirnova%20TD%5BAuthor%5D&cauthor=true&cauthor_uid=19947515). et al. (2009b). [The response of human cancer stem cells on low-dose X-ray exposure]. [Radiats. Biol. Radioecol.](https://www.ncbi.nlm.nih.gov/pubmed/19947515)  49, 528-37. PMID: 19947515

Cancer cells were obtained from patient with breast adenocarcinoma 1 hour after surgery. Cancer stem cells suspension colonies (mammospheres) with CD44+/CD 24-(low) phenotype were obtained from the tumor. The cell population contained 70% of cells with a polyploid set of 1q12 loci [mainly three loci per nucleus, Figure 2S (A)] and 30% of cells with a diploid set of 1q12 loci. After irradiation and cultivation, the cell population was enriched with a diploid 1q12 loci cells (Fig. 2S (A and C). Analysis of the spots distribution in the nuclei with two and three fish signals showed that only cells with a normal 1q12 homologues number have the ability to transpose loci in response to low IR doses (Fig.2S(B)).

The cells with a large f-SatIII repeats amount massively died after irradiation, they were destroyed during MAA fixation and did not attach to the carrier. A significant f-SatIII content increase in the culture medium cell-free DNA was a marker of these cells death.


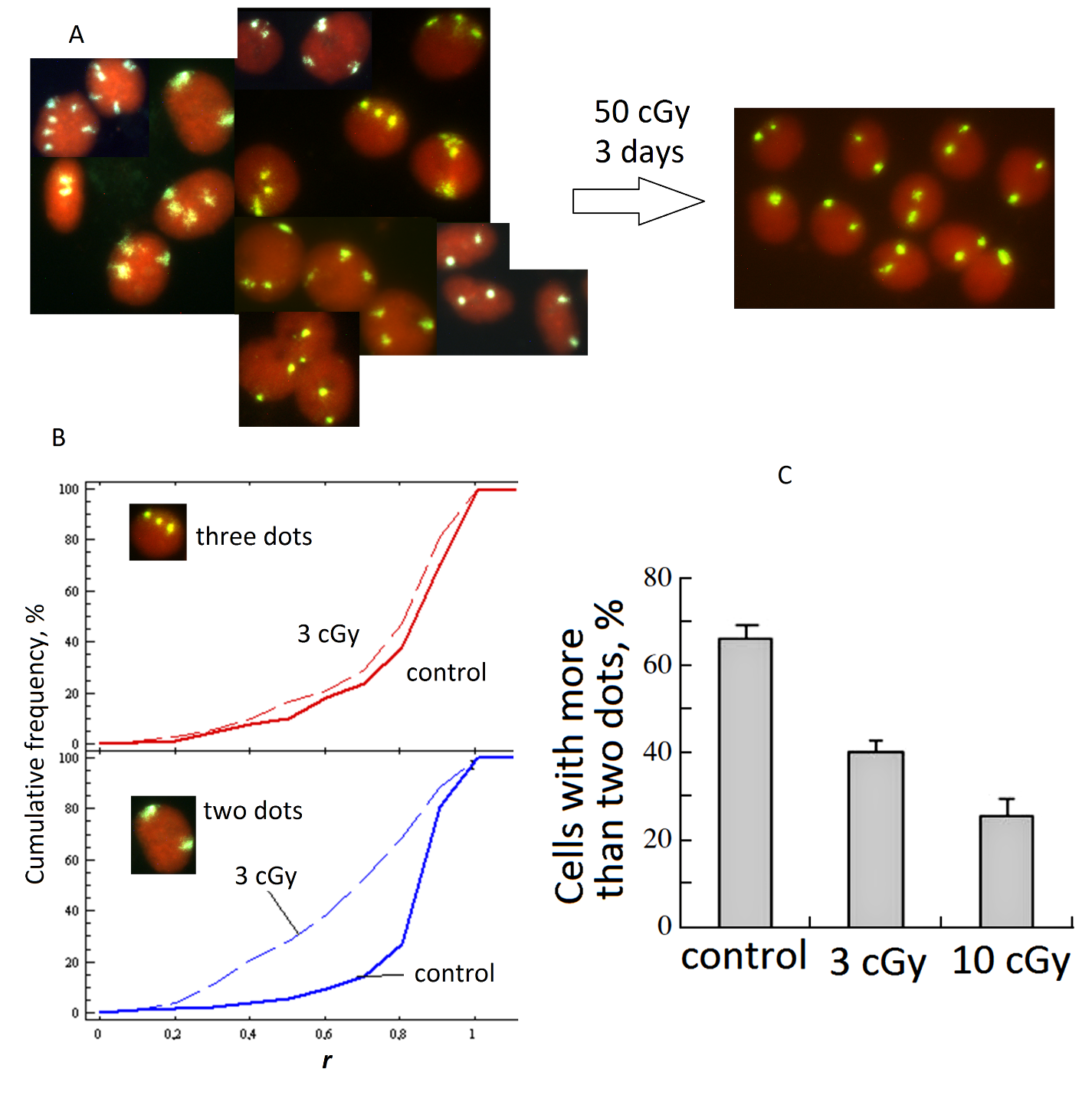


Figure 2S.
